# Intramolecular Hydrogen Bond in Biologically Active *o*-Carbonyl Hydroquinones

**DOI:** 10.3390/molecules19079354

**Published:** 2014-07-03

**Authors:** Maximiliano Martínez-Cifuentes, Boris E. Weiss-López, Leonardo S. Santos, Ramiro Araya-Maturana

**Affiliations:** 1 Laboratorio de Síntesis Asimétrica, Instituto de Química de los Recursos Naturales, Universidad de Talca, Talca, Casilla 747, Chile; E-Mail: lssantos@utalca.cl; 2 Departamento de Química, Facultad de Ciencias, Universidad de Chile, Santiago, Casilla 653, Chile; E-Mail: bweiss@uchile.cl; 3 Departamento de Química Orgánica y Fisicoquímica, Facultad de Ciencias Químicas Y Farmacéuticas, Universidad de Chile, Santiago, Casilla 233, Chile

**Keywords:** hydroquinone, hydrogen bond, DFT, molecular electrostatic potential, natural bond orbital

## Abstract

Intramolecular hydrogen bonds (IHBs) play a central role in the molecular structure, chemical reactivity and interactions of biologically active molecules. Here, we study the IHBs of seven related *o*-carbonyl hydroquinones and one structurally-related aromatic lactone, some of which have shown anticancer and antioxidant activity. Experimental NMR data were correlated with theoretical calculations at the DFT and *ab initio* levels. Natural bond orbital (NBO) and molecular electrostatic potential (MEP) calculations were used to study the electronic characteristics of these IHB. As expected, our results show that NBO calculations are better than MEP to describe the strength of the IHBs. NBO energies (∆E_ij_^(2)^) show that the main contributions to energy stabilization correspond to LP→σ* interactions for IHBs, O_1_^…^O_2_-H_2_ and the delocalization LP→π* for O_2_-C_2_ = C_α(β)_. For the O_1_^…^O_2_-H_2_ interaction, the values of ∆E_ij_^(2)^ can be attributed to the difference in the overlap ability between orbitals i and j (*F*_ij_), instead of the energy difference between them. The large energy for the LP O_2_→π* C_2_ = C_α(β)_ interaction in the compounds 9-Hydroxy-5-oxo-4,8, 8-trimethyl-l,9(8H)-anthracenecarbolactone (**VIII**) and 9,10-dihydroxy-4,4-dimethylanthracen-1(4H)-one (**VII**) (55.49 and 60.70 kcal/mol, respectively) when compared with the remaining molecules (all less than 50 kcal/mol), suggests that the IHBs in **VIII** and **VII** are strongly resonance assisted.

## 1. Introduction

Hydroquinones (HQ) and their oxidized form, quinones (Q), constitute a biologically relevant redox pair. A number of them come from natural sources [1,2] and exhibit a large number of biological activities related to their redox potential [3,4,5,6]. Although *p*-hydroquinone is more stable than *p*-quinone, usually substituted *p*-hydroquinones (*p*-HQ) are thermodynamically less stable than substituted *p*-quinones (*p*-Q), but *p*-Q can be effectively transformed into *p*-HQ by several mechanisms in biological systems [7], and therefore they can co-exist inside living organisms. The biological activity of hydroquinones has been related to their capability to lose an electron followed by deprotonation (or alternatively lose a hydrogen atom), to afford the corresponding semiquinone radical. These intermediates have been associated to biological properties, such as pro-oxidant activity, by interacting with several intracellular molecules, such as DNA and proteins.

Modulation of the electron-transfer capability is very important for the biological activity of quinones and hydroquinones. Among the interactions that play a central role in this issue, the formation of inter- or intramolecular hydrogen bonds in these molecules plays a key role [8,9,10]. A recent electrochemical study about quinones possessing intramolecular hydrogen bonds (IHBs) shows that this interaction stabilizes the anion radical structure, leading to a shift in reduction potentials toward less negative values when compared with quinones without IHBs [11]. IHBs have shown appreciable effects on the antioxidant properties of hydroquinones and related phenols [12,13].

The strength of a hydrogen bond lies between a weak covalent bond and Van der Waals interactions [14], and plays an important role in the geometry of single molecules as well as in the molecular structure of liquids and solids. Hydrogen bonds are important in areas as diverse as biology, chemistry and material science [15]. By definition, a hydrogen bond is an attractive interaction of the X-H^…^Y type, where the molecular fragment X-H acts as a hydrogen bond donor and Y acts as a hydrogen bond acceptor [16].

The *o*-carbonyl hydroquinone moiety is an important structural feature of several natural products with different biological activities, such as doxorubicin, daunorubicin [17], 2,5-dihydroxyacetophenone [18] and peyssonol A [19,20]. *o*-Carbonyl hydroquinones have also been used as building blocks for natural [21,22] and synthetic [23,24,25,26] compounds with a diversity of biological properties. In previous works, our group showed that some *o*-carbonyl hydroquinones can inhibit some tumor cell growth acting at the mitochondrial level [27,28,29]. Also theoretical and experimental NMR studies of some related hydroquinones has been carried out [30]. The IHBs present in these compounds, have been invoked as a key factor for their mitochondrial-mediated anti-cancer activity [31,32].

**Figure 1:**
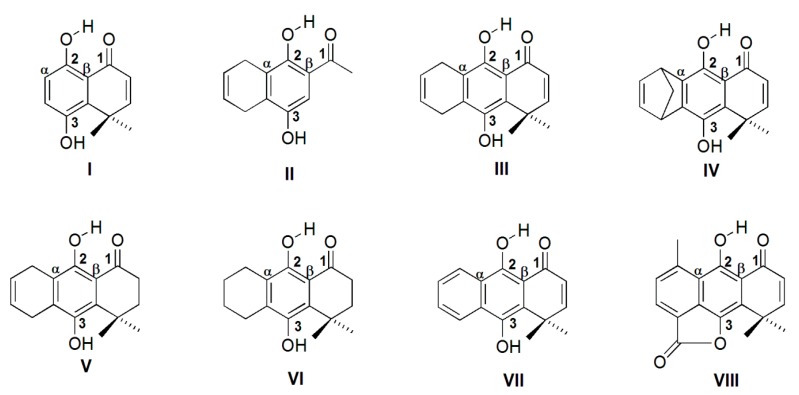
Structure of compounds studied in this work.

On the basis of the above considerations, it appears interesting to study the effect of the molecular structure on the characteristics of the IHBs present in a series of *o*-carbonyl hydroquinones (Figure 1). Therefore, the aim of this work is to study experimentally the IHBs in a series of structurally related *o*-carbonyl hydroquinones and one aromatic lactone, through the use of nuclear magnetic resonance (NMR). Several theoretical approaches can be used to study IHBs, for instance atoms in molecules (AIM) methodology [33,34]. Another scarcely explored methodology is through the use of molecular electrostatic potential (MEP), though this methodology has been mainly used for intermolecular HBs [35,36], more recently it has also been used to study intramolecular HBs [37]. The interesting results from this study made us decide the use this methodology. Besides, we have also used the natural bond orbitals (NBO) methodology, a widely used technique to study IHBs [38].

## 2. Results and Discussion

All the molecules studied here, containing IHBs (Figure 1), can be classified as resonance-assisted hydrogen bonds (RAHBs) [39,40] although this concept has been questioned in recent years [41,42,43,44]. RAHBs are characterized as conjugated molecular fragments connected through the hydrogen bond donor, which provokes a strong hydrogen bond compared with a system without the conjugation. We will take this definition into account in further analysis.

### 2.1. Geometry Optimization

The optimized geometry of all molecules have been obtained at the B3LYP/6-31++G(d,p) and MP2/6-31++G(d,p) levels of theory. The main calculated geometrical parameters for the characterization of IHBs, besides the experimental ^1^H-NMR shifts for H_2_ (Figure 1), are summarized in Table 1. While chloroform is a hydrogen bond donor, it is classified as a weak one (Abraham’s H donor parameter α = 0.15) [45], therefore it does not represent a significant competition to the strong IHBs present in this molecules. Therefore, the ^1^H-NMR chemical shift of H_2_ is a suitable parameter to represent the strength of the IHBs. We also measured the NMR spectra of compound **I**, which is not one of the strongest IHBs in the series, in DMSO-d_6_, a HB acceptor. The chemical shift for H_2_ was 12.55 ppm, only 0.01 ppm away from the value measured in chloroform (12.54 ppm). This observation shows that the studied IHBs remain unchanged, even in DMSO.

**Table 1:** ^1^H-NMR chemical shifts for H_2_ and geometrical parameters for hydrogen bonds calculated at B3LYP/6-31++G(d,p) and MP2/6-31++G(d,p) level of theory. The numbering of compounds is according to Figure 1.

Molecule	δH_2_	B3LYP/6-31++G(d,p)				MP2/6-31++G(d,p)			
		O_1_^…^O_2_	O_2_-H_2_	O_1_^…^H_2_	< O_2_-H_2_^…^O_1_	O_1_^…^O_2_	O_2_-H_2_	O_1_^…^H_2_	< O_2_-H_2_^…^O_1_
**I**	12.54	2.540	0.996	1.638	148	2.573	0.989	1.682	148
**II**	12.32	2.556	0.994	1.657	148	2.592	0.988	1.703	148
**III**	13.08	2.533	0.998	1.624	149	2.567	0.991	1.667	149
**IV**	12.70	2.538	0.996	1.634	149	2.571	0.990	1.679	148
**V**	12.95	2.525	0.997	1.617	149	2.570	0.989	1.676	148
**VI**	12.94	2.521	0.997	1.613	149	2.521	0.997	1.613	149
**VII**	14.53	2.505	1.005	1.584	150	2.543	0.995	1.637	149
**VIII**	15.60	2.482	1.014	1.544	152	2.526	0.999	1.608	150
R^2^		0.89	0.98	0.94	0.92	0.39	0.84	0.50	0.59

Distances in Å, Angle in °, δ in ppm. R^2^ corresponds to correlation between NMR δH_2_ and geometrical parameters.

An inspection to Table 1 shows that the boundary cases are well described by both the MP2 and DFT methods. The largest chemical shift of **VIII** is in agreement with the shortest O_1_^…^H_2_ distance, which indicates the strongest IHB. On the other hand, the lowest chemical shift of **II** accords with the largest O_1_^…^H_2 _distance, showing that **II** has the weakest IHB among these HQs. When all molecules are compared, B3LYP/6-31++G(d,p) calculations are more suitable to describe the IHB geometrical parameters, according with the quantitative correlation of their strength with geometrical parameters and NMR chemical shift data (see correlation coefficients in Table 1). Because B3LYP/6-31++G(d,p) optimized geometries gave better correlations with experimental NMR data, we used these results for further calculations. The main features of the IHBs in this series of molecules, were explored through the use of NBO and MEP calculations.

### 2.2. Molecular Electrostatic Potential

**Figure 2:**
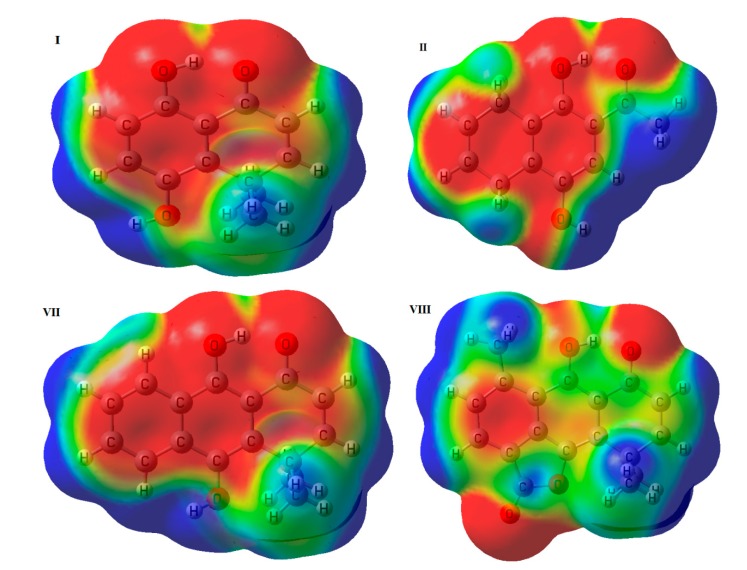
Molecular electrostatic potential (0.004 a.u.) of **I**, **II**, **VII** and **VIII**.

Because the electrostatic characteristic is always present in hydrogen bonds, several methods based in the electrostatic potential have been developed for their study [46,47,48,49]. MEP maps have been used to qualitatively rationalize trends observed in hydrogen bond donors and acceptors [50,51,52]. The MEP maps displayed in Figure 2, were generated projecting the color-coded values onto the 0.004 a.u. iso-potential energy surface. The red color indicates high electron-density sites, blue color indicates low electro-density sites and green-yellow color indicates neutral sites in the molecules. In this Figure, the MEPs of **I** and **II**, which present the weakest IHB, and the MEPs of **VII** and **VIII**, which present the strongest IHB, are shown as representative examples. Electron-rich sites are observed in the aromatic rings. The IHB site shows a remarkably electron-rich zone in both the donor and acceptor oxygens, while a small neutral zone appears on the hydrogen of the IHB from **I**, **II** and **VII**. Unlike the previous cases, **VIII** presents a more extended neutral zone on the hydrogen atom and the electron-rich zone around the oxygens is reduced.

Quantitative MEP descriptors, such as the minimized electrostatic potential (V_min_) and the recently described V_α_(r) parameter, have been used to characterize hydrogen bond basicity and acidity, respectively [53,54,55]. The V_α_(r), calculated for H_2 _at r = 0.55Å, and the value of V_min_ near O_1_ for all molecules are presented in Table 2. From this Table we observe, as a general tendency, that an increase of V_α_(r), and therefore an increase of IHB donor strength, leads to a decrease in V_min_, and therefore lowering the IHB acceptor strength. This trend indicates that structural differences among the molecules, such as the presence of an additional aromatic ring in **VII** and **VIII**, affects both, the donor and acceptor hydrogen bond capabilities. The strong IHB exhibited by **VIII**, is in agreement with the higher value of V_α_(r) (higher acidity of donor) and the lower value for V_min_(O_2_) (higher basicity of acceptor). Nevertheless, considering both parameters separately for all molecules, they do not correlate with the NMR data. In both cases, we could not find a lineal relationship between δH_2 _and V_α_(r) or between δH_2_ and V_min_(O_2_), being R^2^ = 0.37 and R^2^ = 0.05 respectively. This can be explained because V_min_ and the V_α_(r) descriptors are significantly perturbed by the IHB. Regions of positive and negative MEP on the surfaces of hydrogen bond donors and acceptors, are influenced by the formation of intramolecular contacts in these molecules. The trends of V_α_(r) and V_min _with the increase of HB interaction point in the opposite direction, and explain why MEP is not an appropriate descriptor for IHB strength.

**Table 2:** MEP values (B3LYP/6-31+G**//B3LYP/6-31++G**), V_min_ and V_α_(r) (kcal/mol).

Molecule	V_α_(r)	V_min_(O_1_)
**I**	165.0	−48.9
**II**	169.2	−45.0
**III**	163.2	−50.5
**IV**	161.7	−51.3
**V**	165.7	−48.2
**VI**	164.1	−49.2
**VII**	166.2	−49.5
**VIII**	174.9	−43.6

### 2.3. NBO Analysis

The NBO analysis results, natural charges and Wiberg bond orders, are presented in Table 3. Table 4 shows the calculated stabilization energies. Correlations between natural charges and Wiberg bond order (WBO) for the atoms involved in the IHB, and the experimental δH_2_, were studied. The correlations between δH_2_ and natural charge on O_1_, O_2_ and H_2 _gave R^2^ values of 0.81, −0.09 and 0.90, respectively. These results show that the natural charge on the hydrogen atom involved in the IHB is a better parameter than the natural charge on the donor and acceptor oxygens in order to quantify the strength of the IHB. On the other hand, WBO for O_2_-H_2_ and H_2_^…^O_1_ were shown to be excellent parameters for describing the strength of the IHB in this HQ series. In effect, the correlations of δH_2 _with O_2_-H_2 _and with H_2_^…^O_1_ gave R^2^ = 0.99 and R^2^ = 0.98, respectively.

**Table 3:** Natural charges (NC) and Wiberg bond order (WBO) at HF/6-311G** //B3LYP/6-31++G** level for selected atoms in HQs.

Molecule	NC O1	NC O2	NC H2	WBO O2-H2	WBO H2^…^O1
**I**	−0.721	−0.753	0.522	0.6470	0.0699
**II**	−0.717	−0.759	0.524	0.6501	0.0647
**III**	−0.726	−0.765	0.525	0.6395	0.0747
**IV**	−0.727	−0.760	0.522	0.6460	0.0712
**V**	−0.725	−0.763	0.524	0.6393	0.0751
**VI**	−0.726	−0.765	0.523	0.6402	0.0757
**VII**	−0.734	−0.760	0.530	0.6197	0.0890
**VIII**	−0.736	−0.755	0.532	0.6004	0.1051

Analyses of the second order stabilization energies ∆E_ij_^(2)^ (Table 4) allow us to determine the orbital interaction responsible for the IHB. The main hyperconjugative interaction was LPO_1_→σ*O_2_-H_2_. Also, a significant hyperconjugative interaction of type LPO_2_→σ*C_2_=C_α(β)_ which accounts for the delocalization of phenolic oxygen electrons into the aromatic ring, is present. Accordingly, the main contributions to stabilization energy corresponds to the LP→σ* interaction for O_1_^…^O_2_-H_2 _IHB as well as the LP→π* delocalization is the main contribution for the O_2_-C_2_ = C_α(β)_ fragment. It can be noticed that the stabilization energy ∆E_ij_^(2)^ due the IHB formation is higher for **VIII** and **VII**, which present an additional aromatic ring fused to the hydroquinone ring. From the above, it is possible to argue that these IHBs are strongly assisted by resonance, involving the additional ring, which is supported by the high stabilization energy for LP_total(1 + 2)_ O_2_→π* C_2_=C_α(β)_ in **VIII** and **VII** (55.49 and 60.70 Kcal/mol respectively) compared with values found in all remaining molecules, all with less than 50 kcal/mol stabilization energy. It is interesting to compare these results with a recently published work about 1-acylthiourea species, where two conformations with different competing IHB are feasible [56]. It was found that those conformation where IHB was assisted by resonance, presented a stabilization energy corresponding to LP O→σ*H-N around 12 kcal/mol higher than those conformation without resonance assisted IHB.

**Table 4:** Stabilization energies (kcal/mol) for selected NBO pairs (donor-acceptor) given by second order perturbation energies of the Fock matrix in the NBO basis for the HQs (HF/6-311G**//B3LYP/6-31++G**).

Molecule	Φ_i_	Φ_j_	∆E_ij _^(2)^	ε_j_–ε_i_/au	F_ij_/au	Φ_i_	Φ_j_	∆E_ij _^(2)^	ε_j_−ε_i_/au	F_ij_/au
**I**	LP_1_ O_1_	σ* O_2_-H_2_	4.04	1.58	0.072	LP_1_ O_2_	σ* C_2_-C_α_	10.15	1.60	0.114
	LP_2_ O_1_	σ* O_2_-H_2_	28.33	1.18	0.165	LP_2_ O_2_	π* C_2_-C_α_	48.43	0.63	0.168
**II**	LP_1_ O_1_	σ* O_2_-H_2_	3.67	1.58	0.068	LP_1_ O_2_	σ* C_2_-C_α_	9.19	1.61	0.109
	LP_2_ O_1_	σ* O_2_-H_2_	26.02	1.18	1.159	LP_2_ O_2_	π* C_2_-C_α_	48.19	0.64	0.170
**III**	LP_1_ O_1_	σ* O_2_-H_2_	4.08	1.57	0.072	LP_1_ O_2_	σ* C_2_-C_α_	9.42	1.61	0.110
	LP_2_ O_1_	σ* O_2_-H_2_	30.58	1.18	0.171	LP_2_ O_2_	π* C_2_-C_α_	47.57	0.64	0.167
**IV**	LP_1_ O_1_	σ* O_2_-H_2_	4.05	1.58	0.072	LP_1_ O_2_	σ* C_2_-C_α_	10.08	1.58	0.113
	LP_2_ O_1 _	σ* O_2_-H_2_	29.01	1.18	0.167	LP_2_ O_2_	π* C_2_-C_β_	43.47	0.67	0.161
**V**	LP_1_ O_1_	σ* O_2_-H_2_	4.10	1.57	0.072	LP_1_ O_2_	σ* C_2_-C_α_	9.46	1.61	0.110
	LP_2_ O_1_	σ* O_2_-H_2_	30.88	1.18	0.173	LP_2_ O_2_	π* C_2_-C_α_	49.06	0.64	0.170
**VI**	LP_1_ O_1_	σ* O_2_-H_2_	4.15	1.57	0.072	LP_1_ O_2_	σ* C_2_-C_α_	9.67	1.60	0.111
	LP_2_ O_1_	σ* O_2_-H_2_	31.31	1.19	0.174	LP_2_ O_2_	π* C_2_-C_α_	48.22	0.63	0.169
**VII**	LP_1_ O_1_	σ* O_2_-H_2_	4.36	1.54	0.074	LP_1_ O_2_	σ* C_2_-C_α_	9.70	1.63	0.112
	LP_2_ O_1_	σ* O_2_-H_2_	37.44	1.17	0.189	LP_2_ O_2_	π* C_2_-C_α_	55.49	0.65	0.178
**VIII**	LP_1_ O_1_	σ* O_2_-H_2_	4.64	1.51	0.076	LP_1_ O_2_	σ* C_2_-C_α_	9.82	1.60	0.112
	LP_2_ O_1_	σ* O_2_-H_2_	45.67	1.16	0.208	LP_2_ O_2_	π* C_2_-C_α_	60.70	0.64	0.184

A good correlation is observed between the NMR chemical shift of H_2 _and the stabilization energies for LP_total_O_1_→σ* interaction in the O_2_-H_2_ fragment (R^2^ = 0.98). The energy difference between the donor (Φ_i_) and the acceptor (Φ_j_) orbitals, and their overlap, determine the hyperconjugation energy. Lower difference in energy ε_j_–ε_I _as well as high overlap between them (*F*_ij_) favors hyperconjugation. For the LP O_1_→σ* O_2_-H_2 _interaction, ε_j_–ε_I _are very similar for all molecules, so that the difference among ∆E_ij_^(2)^ can be attributed to the difference in the overlap ability between LP O_1 _and σ* O_2_-H_2 _orbitals, given by the *F*_ij_ value (Table 4).

## 3. Experimental

### 3.1. General Information

Melting points were determined on a hot-stage apparatus and are uncorrected. The IR spectra were recorded on a FT-IR Bruker IFS 55 spectrophotometer from KBr discs; wave numbers are reported in cm^−1^. ^1^H-NMR and ^13^C-NMR spectra were obtained from a Bruker DRX-300 spectrometer (300 and 75 MHz, respectively) in CDCl_3_. Chemical shifts were recorded in ppm (δ) relative to TMS as internal standard. *J*-values are given in Hz. Electron impact (IE) high resolution mass spectra were recorded on a Thermo Finnigan model MAT 95XP Mass Spectrometer. Compounds **I**–**V**, **VII** and **VIII** were previously described [57,58,59,60], and new compounds **V** and **VI** were synthesized as follows.

*9,10-Dihydroxy-4,4-dimethyl-3,4,5,8-tetrahydroanthracen-1(2H)-one* (**V**). Butadiene was bubbled through a solution of 8,8-dimethyl-6,7-dihydro-1,4,5(8H)-naphthalenetrione (100 mg, 0.49 mmol) in toluene (10 mL), and the mixture left in a sealed flask at room temperature for a week. Then silica gel (1 g) was added and the mixture stirred overnight at room temperature. The mixture was filtered and the solid washed with dichloromethane. Evaporation of the solvent gave crude of **V**. The pure product (72 mg, 57%) was obtained by column chromatography. ^1^H-NMR δ (CDCl_3_): 1.50 (s, 6H, 2 × CH_3_), 1.93 (t, 2H, *J* = 7 Hz, 3-CH_2_), 2.67 (t, 2H, *J* = 7 Hz, 2-CH_2_), 3.18–3.33 (m, 4H, 5- and 8-CH2), 4.29 (s, 1H, 10-OH), 5.83 (bd, 1H, *J* = 10 Hz, 6- or 7-H), 5.96 (bd, 1H, *J* = 10 Hz, 6- or 7-H), 12.95 (s, 1H, 9-OH). ^13^C-NMRδ (CDCl_3_): 27.21, 23.59, 24.84, 33.85, 34.98, 38.69,121.11, 121.82, 124.52, 142.98, 131.97, 155.23, 205.13. HRMS: *m/z* [M^+^] calcd. For C_16_H_18_O_3_: 258.1256; found: 258.1249. IR (KBr, cm^−1^): 1217, 1614, 2926, 3390. m.p. 130–132 °C.

*9,10-Dihydroxy-4,4-dimethyl-3,4,5,6,7,8-hexahydroanthracen-1(2H)-one* (**VI**). Hydroquinone **VI** was obtained by hydrogenation, stirring a mixture of **III** (100 mg, 0.39 mmol) and 10% Pd/C (25 mg) in ethanol (30 mL), for 4 h under 20 bar of H_2_. The crude product was purified by flash column chromatography, eluting with hexane-ethyl acetate 8:1 yielding pure **VI** (40 mg, 38%). ^1^H-NMR δ (CDCl_3_): 1.50 (s, 6H, 2 CH_3_), 1.69–1.87 (m, 4H, 6- and 7-CH_2_), 1.91 (t, 2H, *J* = 7 Hz, 3-CH_2_), 2.57 (t, 2H, *J* = 7 Hz, 2-CH2), 2.65 (t, 4H, *J* = 7 Hz, 5- and 8- CH2), 4.38 (s, 1H, 10-OH), 12.94 (s, 1H, 9-OH). HRMS: *m/z* [M^+^] calcd. For C_16_H_20_O_3_: 260.14124; found: 260.14045. IR(KBr): 1610, 2927, 3308 cm^−1^. m.p. 192–193.5 °C.

### 3.2. Theoretical Methods

The calculations were carried out using the Gaussian03 [61] program package, running in a Microsystem cluster of blades. Geometries were optimized at Møller-Plesset second-order-corrected [62] (MP2) ab-initio level, and Becke three-parameter Lee-Yang-Parr [63] (B3LYP) density functional theory (DFT) level. 6-31++G** basis set was used in both cases. We carried out the calculation in vacuum because this model is commonly associated to aprotic non-polar solvents, like chloroform and because the energies of molecules in both models are very similar [64]. No imaginary vibrational frequencies were found at the optimized molecular geometries, which indicate that they are true minima of the potential energy surface. The theoretical study of intramolecular hydrogen bond was carried out through the calculation of MEP and a NBO analysis.

The MEP is related to the electron density and it has been widely used to study hydrogen bonds [51], reactivity [65], and to correlate biological activity with molecular structure [66,67]. The MEP minimum (V_min_) is computed from the optimized geometries, using equation 1 at the B3LYP/6-31+G(d,p) level of theory, which has been described as adequate for this kind of calculations [55]:

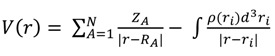
(1)
here Z_A_ is the nuclear charge and ρ(r) the electron density. V_min_ has been described [53,54] as a useful predictor of hydrogen bond acceptor basicity. Recently, it has been proposed that the V_α_(r) descriptor [55], calculated at a distance of 0.55 Å from the hydrogen atom along the O-H bond, is also useful to predict hydrogen bond donor acidity.

On the other hand, the NBO method has been recognized as a powerful tool to gain insights into orbital interactions, such as stabilization energies caused by electron transfer and hyperconjugation stabilization energies [68,69]. The NBOs are one of the consequences of natural localized orbital sets that include natural atomic (NAO), hybrid (NHO) and semi-localized molecular orbital (NLMO) sets, intermediates between basis atomic orbitals (AOs) and canonical molecular orbitals (MOs) [34]. The NBO method involves population analysis, which distributes computed electron density into orbitals in the way chemist think, in terms of physical organic chemistry. The interaction between filled and antibonding orbitals represents the deviation of the molecule from the Lewis structure and can be used as a measure of delocalization due to the presence of hydrogen bonding interaction [34]. These hyperconjugative interactions play an important role in hydrogen bonding. The donor-acceptor interaction (stabilization energy) can be calculated with second-order perturbation theory analysis. The hiperconjugative interaction between lone pair (LP) on acceptor oxygen and sigma antibonding on donor H-O (LP_O_→σ*_H-O__’_) in O^…^H-O’ complex, has been described as a major contribution to hydrogen bond interaction obtained by NBO analysis [70,71]. The NBO calculations were carried out at HF/6-311++G(d,p) level. The change of DFT to *ab initio* methods for NBO calculation has been described previously, to avoid possible unphysical results previously found when DFT method is used [72,73].

## 4. Conclusions

Differences in molecular structures among the members of this series have significant influence on the characteristics of IHB C-O^…^H-O they present. The structures of these molecules were calculated using DFT and *ab initio* MO calculations, and contrasted with experimental data from ^1^H-NMR chemical shifts. The quantitative correlation between calculated geometrical parameters and ^1^H-NMR chemical shift in these IHBs was better described by DFT than *ab initio* molecular orbital calculations.

Maps of molecular electrostatic potential (MEP) showed a large negative area on the oxygen and a small neutral area on the hydrogen of the C-O^…^H-O fragment. The neutral zone increased remarkably in structure **VIII**, which possess the strongest IHB. Quantitatively, MEP descriptors V_α_(r) and V_min_ exhibit a general tendency, where the increasing of IHB donor strength (reflected by V_α_(r) leads to a decrease in the IHB acceptor strength (reflect by V_min_), but they do not correlate well with the ^1^H-NMR data. Natural bond orbital (NBO) analysis shows that in our case, Wiberg bond order is a better descriptor of IHB strength than natural charges.

Analyses of the second order stabilization NBO energies (∆E_ij_^(2)^) shows that the main contributions to stabilization energy correspond to LP→σ* interactions for IHB O_1_^…^O_2_-H_2 _and the delocalization LP→π* for O_2_-C_2_ = C_α__(β__)_. The NMR chemical shift of H_2 _correlates well with the stabilization energies for LP_total_O_2_→σ* O_1_-H_1_. For the above interaction, the difference in ∆E_ij_^(2) ^among the molecules can be attributed to the difference in the overlapping (*F*_ij_) ability between LP O_1_ and σ* O_2_-H_2_ orbitals, instead of the orbitals energy differences (ε_j_–ε_I_). The large energy for LP O_2_→π* C_2_ = C_α(β)_ in **VIII** and **VII** (55.49 and 60.70 kcal/mol, respectively), compared with the remaining molecules (all values less than 50 kcal/mol), suggests that the IHBs in **VIII** and **VII** are strongly resonance assisted hydrogen bonds.

In view of results of MEP and NBO calculations, we note that the latter provide a better quantitative description of the strength of IHBs in these molecules, and is more suitable to understand and predict the characteristics of this interaction. These results not only might be of interest to gain insight into intramolecular hydrogen bonds but also can help to rationalize the design of new hydroquinones with biological activity.

## Supplementary Materials

Cartesian coordinates and energies for the calculated optimized structures. Supplementary Materials can be accessed at: http://www.mdpi.com/1420-3049/19/7/9354/s1.
