# Supplementary material for: Intramolecular Hydrogen Bond in Biologically Active o-Carbonyl Hydroquinones
Source: Molecules. 2014 Jul 3;19(7):9354–68. doi: 10.3390/molecules19079354 (PMC6270916; doi:10.3390/molecules19079354)
Supplement: Supplementary file 1 [file molecules-19-09354-s001.pdf]

# Supporting Information

**Optimized geometries and absolute energies.**

B3LYP/6−31++G(d,p)

Compound **I**

E(RB3LYP) = −690.22332819 a.u.

| | | | |
|---|---|---|---|
| C | 0.45727800 | 2.57290900 | −0.00002100 |
| C | −0.63595400 | 1.69602800 | −0.00008900 |
| C | −0.44590500 | 0.30737100 | −0.00010400 |
| C | 0.89592500 | −0.17395600 | −0.00004400 |
| C | 1.99782000 | 0.72589300 | 0.00001100 |
| C | 1.76132000 | 2.10486300 | 0.00002900 |
| H | 0.27458500 | 3.64605000 | 0.00000700 |
| C | 1.17817900 | −1.61774900 | −0.00009100 |
| H | 2.60715400 | 2.78340300 | 0.00008300 |
| C | 0.04921600 | −2.54604800 | −0.00007100 |
| C | −1.21495800 | −2.10065400 | −0.00016900 |
| H | 0.29546300 | −3.60331400 | −0.00010700 |
| H | −2.03529300 | −2.81736300 | −0.00034600 |
| O | 3.27496700 | 0.30323700 | 0.00004200 |
| H | 3.24572300 | −0.69230800 | 0.00000500 |
| O | −1.92367100 | 2.20224600 | −0.00006900 |
| H | −1.88811500 | 3.16712100 | −0.00016000 |
| O | 2.34716700 | −2.06177800 | −0.00007000 |
| C | −1.63794600 | −0.65681200 | −0.00000400 |
| C | −2.51241800 | −0.45341200 | −1.27356100 |
| H | −1.91888800 | −0.61522800 | −2.17851500 |
| H | −3.34097600 | −1.17035000 | −1.27522700 |
| H | −2.92734000 | 0.55473100 | −1.30007000 |
| C | −2.51176400 | −0.45358900 | 1.27402000 |
| H | −1.91778900 | −0.61559700 | 2.17866000 |
| H | −2.92659400 | 0.55458900 | 1.30094900 |
| H | −3.34039400 | −1.17044700 | 1.27605600 |

Compound **II**

E(RB3LYP) = −690.22456699 a.u.

| | | | |
|---|---|---|---|
| C | −2.30527700 | −1.03804500 | −0.00038100 |
| O | 0.04894400 | −2.64639200 | 0.00048600 |
| H | 0.89883800 | −3.10173400 | 0.00135300 |
| O | 0.88380700 | 2.80687700 | 0.00066400 |
| H | −0.00829600 | 3.25381600 | 0.00032300 |
| C | −2.55066800 | −1.90080300 | −1.27397100 |
| H | −3.57701700 | −2.28477800 | −1.27256700 |
| H | −2.41266000 | −1.30013600 | −2.17826800 |
| H | −1.86442300 | −2.74748900 | −1.30584000 |
| H | −1.86557000 | −2.74716700 | 1.30623700 |
| C | −2.55130200 | −1.90005200 | 1.27405200 |
| H | −3.57778800 | −2.28360700 | 1.27236400 |
| H | −2.41288500 | −1.29887800 | 2.17788700 |
| O | −1.62782800 | 3.13546700 | −0.00030700 |
| C | 3.13836400 | 1.25189300 | 0.00109000 |
| H | 3.23094000 | 1.92476600 | −0.86373200 |
| H | 3.23229300 | 1.92108800 | 0.86868300 |
| C | 2.72559900 | −1.72003900 | 0.00131600 |
| H | 2.65955600 | −2.38283100 | 0.88162100 |
| H | 2.65754000 | −2.38704500 | −0.87559900 |
| C | −1.79840400 | 1.89511600 | −0.00036400 |
| C | −3.15466700 | 1.34952600 | −0.00067100 |
| H | −3.96480800 | 2.07216600 | −0.00078500 |
| C | −3.36933000 | 0.02649100 | −0.00069300 |
| H | −4.39153400 | −0.35014400 | −0.00092600 |
| C | 4.26083600 | 0.25739700 | −0.00186600 |
| H | 5.27138600 | 0.66063700 | −0.00419400 |
| C | 4.08066500 | −1.06503200 | −0.00192600 |
| H | 4.93840200 | −1.73395200 | −0.00432500 |
| C | 1.76295200 | 0.62638700 | 0.00075100 |
| C | 1.55643700 | −0.75344500 | 0.00063000 |
| C | 0.64555400 | 1.47940100 | 0.00037400 |
| C | −0.67073100 | 0.95476300 | −0.00004300 |
| C | −0.88780500 | −0.45224100 | −0.00007900 |
| C | 0.24221600 | −1.27503900 | 0.00028800 |

Compound **III**

E(RB3LYP) = −845.06112225 a.u.

| | | | |
|---|---|---|---|
| C | −2.30527700 | −1.03804500 | −0.00038100 |
| O | 0.04894400 | −2.64639200 | 0.00048600 |
| H | 0.89883800 | −3.10173400 | 0.00135300 |
| O | 0.88380700 | 2.80687700 | 0.00066400 |
| H | −0.00829600 | 3.25381600 | 0.00032300 |
| C | −2.55066800 | −1.90080300 | −1.27397100 |
| H | −3.57701700 | −2.28477800 | −1.27256700 |
| H | −2.41266000 | −1.30013600 | −2.17826800 |
| H | −1.86442300 | −2.74748900 | −1.30584000 |
| H | −1.86557000 | −2.74716700 | 1.30623700 |
| C | −2.55130200 | −1.90005200 | 1.27405200 |
| H | −3.57778800 | −2.28360700 | 1.27236400 |
| H | −2.41288500 | −1.29887800 | 2.17788700 |
| O | −1.62782800 | 3.13546700 | −0.00030700 |
| C | 3.13836400 | 1.25189300 | 0.00109000 |
| H | 3.23094000 | 1.92476600 | −0.86373200 |
| H | 3.23229300 | 1.92108800 | 0.86868300 |
| C | 2.72559900 | −1.72003900 | 0.00131600 |
| H | 2.65955600 | −2.38283100 | 0.88162100 |
| H | 2.65754000 | −2.38704500 | −0.87559900 |
| C | −1.79840400 | 1.89511600 | −0.00036400 |
| C | −3.15466700 | 1.34952600 | −0.00067100 |
| H | −3.96480800 | 2.07216600 | −0.00078500 |
| C | −3.36933000 | 0.02649100 | −0.00069300 |
| H | −4.39153400 | −0.35014400 | −0.00092600 |
| C | 4.26083600 | 0.25739700 | −0.00186600 |
| H | 5.27138600 | 0.66063700 | −0.00419400 |
| C | 4.08066500 | −1.06503200 | −0.00192600 |
| H | 4.93840200 | −1.73395200 | −0.00432500 |
| C | 1.76295200 | 0.62638700 | 0.00075100 |
| C | 1.55643700 | −0.75344500 | 0.00063000 |
| C | 0.64555400 | 1.47940100 | 0.00037400 |
| C | −0.67073100 | 0.95476300 | −0.00004300 |
| C | −0.88780500 | −0.45224100 | −0.00007900 |
| C | 0.24221600 | −1.27503900 | 0.00028800 |

Compound**IV**

E(RB3LYP) = −883.12882130 a.u.

| | | | |
|---|---|---|---|
| O | −1.81194500 | 3.11747500 | 0.04999100 |
| O | 0.71017000 | 2.86503400 | −0.08706500 |
| H | −0.18939800 | 3.28981900 | −0.03493900 |
| O | −0.02735800 | −2.65942700 | −0.10552300 |
| H | 0.84190800 | −3.07694800 | −0.14691100 |
| C | 3.50895000 | −0.95522600 | 1.07444500 |
| H | 3.73230700 | −1.65684500 | 1.86989300 |
| C | 3.67821300 | 0.37410400 | 1.07543700 |
| H | 4.07263400 | 0.99191400 | 1.87376900 |
| C | 1.58411800 | 0.67299900 | −0.17645300 |
| C | 1.39757400 | −0.70982800 | −0.18099500 |
| C | 0.50050900 | 1.53298200 | −0.09676800 |
| C | −0.80953500 | 0.95833800 | −0.03074500 |
| C | −1.00498900 | −0.45355100 | −0.03364000 |
| C | 0.13266900 | −1.28341500 | −0.10516300 |
| C | −3.30156000 | 1.30497200 | 0.11277800 |
| H | −4.12134000 | 2.01469100 | 0.16569900 |
| C | −3.49419100 | −0.02080700 | 0.11002300 |
| H | −4.50770300 | −0.41672800 | 0.16265600 |
| C | −1.95624000 | 1.87394300 | 0.04421000 |
| C | 3.09534500 | 0.91969900 | −0.24150900 |
| H | 3.39837100 | 1.92479000 | −0.53096100 |
| C | 3.47626100 | −0.26724400 | −1.18150900 |
| H | 3.00939000 | −0.20322600 | −2.16944600 |
| H | 4.55617500 | −0.41213100 | −1.27550800 |
| C | 2.80812600 | −1.32647300 | −0.24778500 |
| H | 2.88196500 | −2.37365700 | −0.55310700 |
| C | −2.41123600 | −1.06221600 | 0.03991600 |
| C | −2.70898900 | −1.91846500 | −1.22775600 |
| H | −2.63014600 | −1.30703600 | −2.13190800 |
| H | −2.01128900 | −2.75296000 | −1.30465000 |
| H | −3.72683900 | −2.32085800 | −1.17500200 |
| C | −2.56735300 | −1.94241200 | 1.31641900 |
| H | −3.58432100 | −2.34719000 | 1.36735300 |
| H | −1.86376400 | −2.77523300 | 1.30069800 |
| H | −2.39092400 | −1.34745400 | 2.21770800 |

Compound **V**

E(RB3LYP) = −846.28279889 a.u.

| | | | |
|---|---|---|---|
| C | −2.25800000 | −1.08847600 | −0.09268000 |
| O | 0.12797200 | −2.64852200 | 0.15836100 |
| H | 0.97942300 | −3.09753900 | 0.10370900 |
| O | 0.90525500 | 2.80097000 | −0.08938900 |
| H | 0.01121400 | 3.24276200 | −0.06688900 |
| C | −2.37175200 | −2.18483700 | −1.18220800 |
| H | −3.42542400 | −2.46689800 | −1.29495100 |
| H | −2.02116100 | −1.80819900 | −2.14954400 |
| H | −1.80120200 | −3.07821900 | −0.93455100 |
| H | −1.90946600 | −2.47549300 | 1.56562400 |
| C | −2.62181700 | −1.69631600 | 1.28555100 |
| H | −3.62202300 | −2.14396000 | 1.24599400 |
| H | −2.62045400 | −0.93927300 | 2.07679500 |
| O | −1.59660100 | 3.11051100 | 0.04781300 |
| C | 3.17399800 | 1.27266700 | −0.07477700 |
| H | 3.25772600 | 1.90422300 | −0.97110700 |
| H | 3.26433500 | 1.98309700 | 0.75976800 |
| C | 2.78903600 | −1.69716800 | 0.09618400 |
| H | 2.72933800 | −2.29661100 | 1.02052500 |
| H | 2.72518800 | −2.42526300 | −0.73153800 |
| C | −1.75967500 | 1.87512200 | 0.07899000 |
| C | 4.30606100 | 0.28979100 | −0.03472600 |
| H | 5.31262400 | 0.70147600 | −0.06873400 |
| C | 4.13801000 | −1.03211000 | 0.04055100 |
| H | 5.00171800 | −1.69272400 | 0.06806800 |
| C | 1.80510500 | 0.63365800 | −0.04096300 |
| C | 1.61080500 | −0.74427300 | 0.03217800 |
| C | 0.67715200 | 1.47330200 | −0.06237200 |
| C | −0.63752200 | 0.93698400 | −0.03719200 |
| C | −0.84139200 | −0.47837400 | −0.03191300 |
| C | 0.29974600 | −1.28033300 | 0.04622500 |
| C | −3.29374500 | −0.00436700 | −0.47952700 |
| H | −3.20049800 | 0.20773100 | −1.55289100 |
| H | −4.29963600 | −0.41324300 | −0.33006200 |
| C | −3.13785900 | 1.30575700 | 0.28677500 |
| H | −3.86878900 | 2.05881400 | −0.02110000 |
| H | −3.27483200 | 1.14948600 | 1.36602500 |

Compound **VI**

E(RB3LYP) = −847.50719150 a.u.

| | | | |
|---|---|---|---|
| C | 2.27878400 | −1.10237600 | 0.15101800 |
| O | −0.07525500 | −2.64318200 | −0.34606800 |
| H | −0.92711700 | −3.06588900 | −0.50689900 |
| O | −0.83319400 | 2.82752700 | −0.07307100 |
| H | 0.06521000 | 3.25404800 | 0.00137500 |
| C | 2.28667600 | −2.22219100 | 1.22278600 |
| H | 3.32415200 | −2.51441800 | 1.42504200 |
| H | 1.85164900 | −1.86367100 | 2.16230300 |
| H | 1.73667700 | −3.10647300 | 0.90576000 |
| H | 2.07424800 | −2.45533800 | −1.55861000 |
| C | 2.76168200 | −1.68563300 | −1.20133400 |
| H | 3.75243400 | −2.13986400 | −1.08091500 |
| H | 2.83405600 | −0.91364300 | −1.97450800 |
| O | 1.67062800 | 3.10280100 | 0.03890500 |
| C | −3.14850200 | 1.19211100 | −0.31629900 |
| H | −3.16232900 | 2.25923000 | −0.08731600 |
| H | −3.50937300 | 1.09093100 | −1.35107300 |
| C | −2.81194500 | −1.54746000 | −0.45688400 |
| H | −3.19626200 | −1.41580300 | −1.47868300 |
| H | −2.64255600 | −2.62512300 | −0.33988300 |
| C | 1.82196500 | 1.86594500 | −0.00246500 |
| C | −1.74061700 | 0.65867900 | −0.22840000 |
| C | −1.55210100 | −0.72336900 | −0.29814100 |
| C | −0.62403600 | 1.49628000 | −0.09501600 |
| C | 0.68394600 | 0.93932600 | −0.00695000 |
| C | 0.87691300 | −0.47820800 | −0.02296800 |
| C | −0.25572600 | −1.27795200 | −0.21936600 |
| C | 3.28481800 | −0.03803400 | 0.65239900 |
| H | 3.09715500 | 0.15335500 | 1.71726900 |
| H | 4.29626300 | −0.45495200 | 0.58521700 |
| C | 3.20876500 | 1.28774400 | −0.09723500 |
| H | 3.91424200 | 2.02789400 | 0.29088800 |
| H | 3.44130700 | 1.15250700 | −1.16285800 |
| C | −3.90077900 | −1.11981300 | 0.55754100 |
| H | −4.83935200 | −1.61732000 | 0.28961600 |
| H | −3.61673000 | −1.50354100 | 1.54340200 |
| C | −4.09465700 | 0.42032400 | 0.63005400 |
| H | −5.13087800 | 0.68673100 | 0.39495100 |
| H | −3.91134500 | 0.76192900 | 1.65434000 |

Compound **VII**

E(RB3LYP) = −843.87349516 a.u.

| | | | |
|---|---|---|---|
| C | −4.03846400 | −1.11479000 | −0.04046600 |
| C | −2.76026500 | −1.63911000 | −0.05252300 |
| C | −1.61827900 | −0.79386000 | −0.00917000 |
| C | −1.83504200 | 0.61604300 | 0.01681200 |
| C | −3.15481300 | 1.13181900 | 0.02958000 |
| C | −4.24191900 | 0.28235900 | 0.00838700 |
| C | −0.27695600 | −1.30095600 | 0.01136900 |
| C | −0.70316900 | 1.48676500 | 0.01388600 |
| C | 0.59936200 | 0.95990800 | −0.00635900 |
| C | 0.82510200 | −0.46584300 | 0.00127800 |
| C | 2.25422300 | −1.03084000 | 0.00207500 |
| C | 3.30472100 | 0.04807000 | −0.02109600 |
| C | 3.07909400 | 1.36914700 | −0.03251900 |
| C | 1.71950700 | 1.90336300 | −0.01875600 |
| H | −4.89291100 | −1.78412300 | −0.07902800 |
| H | −2.65004000 | −2.71755300 | −0.12721000 |
| H | −3.28522000 | 2.20762400 | 0.05345400 |
| H | −5.25056100 | 0.68351800 | 0.01881500 |
| H | 4.33082800 | −0.31772900 | −0.02823100 |
| H | 3.88357600 | 2.09782600 | −0.04808800 |
| O | −0.07775700 | −2.67013000 | 0.01070100 |
| H | −0.86172500 | −3.11391000 | 0.35612900 |
| O | −0.94080600 | 2.80442600 | 0.02519600 |
| H | −0.03959300 | 3.24966000 | 0.01248400 |
| C | 2.52064700 | −1.86907600 | 1.28748100 |
| H | 3.55489900 | −2.23141000 | 1.28970300 |
| H | 2.37108000 | −1.25912000 | 2.18363200 |
| H | 1.85329800 | −2.73018600 | 1.33071900 |
| H | 1.83430300 | −2.76730700 | −1.27630100 |
| C | 2.50628000 | −1.90874200 | −1.25952700 |
| H | 3.53872100 | −2.27637700 | −1.25808400 |
| H | 2.35350300 | −1.32454200 | −2.17213000 |
| O | 1.54102100 | 3.14546500 | −0.01571700 |

Compound **VIII**

E(RB3LYP) = −995.33124708 a.u.

| | | | |
|---|---|---|---|
| C | 3.59857300 | −0.89359100 | −0.00010800 |
| C | 2.28888400 | −1.33804500 | −0.00006200 |
| C | 1.25504100 | −0.39580600 | −0.00008600 |
| C | 1.43219800 | 0.99448200 | −0.00006000 |
| C | 2.78695900 | 1.45812600 | −0.00009700 |
| C | 3.81490900 | 0.50273300 | −0.00012900 |
| C | 0.02051400 | −1.06260400 | −0.00015700 |
| C | 0.20456900 | 1.74325200 | 0.00005000 |
| H | 4.43681000 | −1.58350400 | −0.00008600 |
| H | 4.83935300 | 0.86338200 | −0.00018300 |
| O | 0.24499000 | −2.43656200 | 0.00004400 |
| O | 0.26780500 | 3.07508800 | 0.00032600 |
| H | −0.69194600 | 3.40185700 | 0.00042900 |
| C | −2.65421700 | −1.98118700 | −1.26691200 |
| H | −3.63124400 | −2.47648600 | −1.27480500 |
| H | −2.56526900 | −1.38251800 | −2.17832300 |
| H | −1.87853700 | −2.75000000 | −1.27428100 |
| H | −1.87780300 | −2.74482500 | 1.28030000 |
| C | −2.65484700 | −1.97734800 | 1.26933600 |
| H | −3.63094800 | −2.47447000 | 1.27696000 |
| H | −2.56857900 | −1.37593400 | 2.17918300 |
| O | −2.21359300 | 3.14245000 | 0.00037300 |
| C | 1.64377600 | −2.66810200 | 0.00005400 |
| O | 2.08884000 | −3.78686700 | 0.00012600 |
| C | 3.13915800 | 2.92323500 | −0.00015400 |
| H | 2.71962100 | 3.43062300 | −0.87410100 |
| H | 2.72053200 | 3.43041100 | 0.87437900 |
| H | 4.22360700 | 3.05749600 | −0.00065600 |
| C | −2.25945800 | 1.88666400 | −0.00020100 |
| C | −3.56174300 | 1.21833600 | −0.00116600 |
| H | −4.42925700 | 1.87090000 | −0.00188500 |
| C | −3.67095300 | −0.11846100 | −0.00158300 |
| H | −4.65984600 | −0.57515800 | −0.00275800 |
| C | −1.16738800 | −0.37963900 | −0.00031600 |
| C | −1.04752500 | 1.06767200 | −0.00017400 |
| C | −2.52025600 | −1.08715800 | −0.00008700 |

MP2/6−31++G(d,p)

Compound **I**

E(MP2) = −688.20266692a.u.

| | | | |
|---|---|---|---|
| C | 0.42855200 | 2.57543400 | −0.01876000 |
| C | −0.65134100 | 1.68470200 | 0.00355300 |
| C | −0.44841200 | 0.29495800 | −0.01081700 |
| C | 0.89673600 | −0.17009100 | 0.03189900 |
| C | 1.98659800 | 0.73839000 | −0.00755400 |
| C | 1.73778000 | 2.11275700 | 0.00888900 |
| H | 0.23673800 | 3.64409000 | −0.01329700 |
| C | 1.18386800 | −1.61400400 | −0.00331900 |
| H | 2.57720800 | 2.79547800 | −0.00808200 |
| C | 0.05901400 | −2.54407800 | −0.01029900 |
| C | −1.21063800 | −2.09794600 | −0.00989600 |
| H | 0.30356200 | −3.59906900 | −0.01959200 |
| H | −2.02766000 | −2.81539900 | −0.01494900 |
| O | 3.28380100 | 0.34012600 | 0.00540900 |
| H | 3.27091200 | −0.64836600 | −0.00498200 |
| O | −1.94868200 | 2.16941600 | −0.00639700 |
| H | −1.91480900 | 3.13635800 | −0.00241400 |
| O | 2.35773900 | −2.06080600 | −0.01156000 |
| C | −1.62932900 | −0.66141700 | 0.00103900 |
| C | −2.50846800 | −0.45572200 | −1.25363700 |
| H | −1.91599300 | −0.61565100 | −2.15609400 |
| H | −3.32936000 | −1.17660100 | −1.24723200 |
| H | −2.92539300 | 0.54802700 | −1.27499400 |
| C | −2.47380000 | −0.46201700 | 1.28026900 |
| H | −1.85641300 | −0.62389400 | 2.16542400 |
| H | −2.89016500 | 0.54197100 | 1.31421300 |
| H | −3.29485700 | −1.18262800 | 1.29418100 |

Compound **II**

E(MP2) = −688.19562204 a.u.

| | | | |
|---|---|---|---|
| C | 3.65661900 | −0.17111700 | −0.19748100 |
| C | 1.30024100 | 0.64572600 | 0.04887800 |
| C | 0.85783800 | −0.67837600 | 0.09705900 |
| C | 3.23080800 | −1.44392000 | −0.18631200 |
| H | 4.69541000 | 0.04519500 | −0.42248900 |
| C | 0.34919400 | 1.68705000 | 0.03907200 |
| C | −0.52330800 | −0.96055800 | 0.03998300 |
| H | 3.93048700 | −2.24372400 | −0.40429500 |
| C | −1.47425500 | 0.08460500 | 0.03820000 |
| C | −1.00891900 | 1.41829900 | −0.02489400 |
| H | −1.71547800 | 2.23996400 | −0.03633900 |
| C | 1.81783200 | −1.83912600 | 0.12452700 |
| H | 1.47145500 | −2.59793300 | −0.58190300 |
| H | 1.76853000 | −2.32421600 | 1.10740800 |
| C | 2.76393900 | 0.99595500 | 0.10608800 |
| H | 2.95903900 | 1.81471000 | −0.59146000 |
| H | 2.99867200 | 1.40295500 | 1.09781800 |
| C | −2.91027300 | −0.22317000 | −0.03811200 |
| O | −3.31884000 | −1.40560800 | −0.04003000 |
| C | −3.91847700 | 0.89871800 | −0.09891700 |
| H | −3.85486700 | 1.52041600 | 0.79511600 |
| H | −3.73929300 | 1.53380100 | −0.96681500 |
| H | −4.91115500 | 0.46181400 | −0.16638700 |
| O | −0.87866400 | −2.27208100 | 0.06964800 |
| H | −1.86538800 | −2.28983600 | 0.02825000 |
| O | 0.85875400 | 2.97148300 | 0.02337900 |
| H | 0.12514100 | 3.60197400 | 0.02857400 |

Compound**III**

E(MP2) = −842.55329444 a.u.

| | | | |
|---|---|---|---|
| C | −2.29112000 | −1.04119100 | −0.00865700 |
| O | 0.04072600 | −2.64429800 | 0.02289100 |
| H | 0.89609400 | −3.09169100 | −0.02614500 |
| O | 0.90067100 | 2.81232400 | 0.02642600 |
| H | 0.01771100 | 3.26158600 | 0.00475300 |
| C | −2.51554500 | −1.91904100 | −1.26131300 |
| H | −3.53808700 | −2.30416700 | −1.26037000 |
| H | −2.37183000 | −1.32399100 | −2.16491600 |
| H | −1.82538800 | −2.75874800 | −1.27439100 |
| H | −1.84442700 | −2.71432800 | 1.32035500 |
| C | −2.53016100 | −1.87170100 | 1.27319600 |
| H | −3.55442200 | −2.25226000 | 1.27929300 |
| H | −2.38860900 | −1.24530800 | 2.15563000 |
| O | −1.64477000 | 3.13667200 | −0.02521100 |
| C | 3.12263500 | 1.25543600 | 0.03910900 |
| H | 3.17047700 | 1.97552500 | −0.78393600 |
| H | 3.24633800 | 1.86073800 | 0.94454000 |
| C | 2.71777900 | −1.72043100 | 0.04660200 |
| H | 2.68569800 | −2.29547100 | 0.98281900 |
| H | 2.62283900 | −2.45488100 | −0.76586300 |
| C | −1.80468300 | 1.88953900 | −0.01435800 |
| C | −3.15544600 | 1.33645600 | −0.03545100 |
| H | −3.97076100 | 2.04910300 | −0.05703900 |
| C | −3.36017400 | 0.00622200 | −0.03583300 |
| H | −4.37820300 | −0.37579300 | −0.05199500 |
| C | 4.23951000 | 0.26609000 | −0.07421300 |
| H | 5.24498900 | 0.66431800 | −0.16011000 |
| C | 4.06146300 | −1.06314600 | −0.07568300 |
| H | 4.91962100 | −1.72137700 | −0.15759300 |
| C | 1.75634000 | 0.62487900 | 0.05282100 |
| C | 1.55421900 | −0.75767400 | 0.00031700 |
| C | 0.64248200 | 1.47673500 | 0.00965200 |
| C | −0.67202800 | 0.95494600 | 0.04228200 |
| C | −0.89160700 | −0.44874700 | −0.01070100 |
| C | 0.24215700 | −1.27351300 | 0.02258500 |

Compound**IV**

E(MP2) = −880.52521607a.u.

| | | | |
|---|---|---|---|
| O | −1.82615100 | 3.11925800 | 0.02966500 |
| O | 0.73151400 | 2.87211200 | −0.07087400 |
| H | −0.15847600 | 3.30337000 | −0.03559500 |
| O | −0.03206500 | −2.66000700 | −0.11517700 |
| H | 0.84674200 | −3.06492000 | −0.12891400 |
| C | 3.47593200 | −0.97077500 | 1.06206900 |
| H | 3.68776600 | −1.67616700 | 1.85400300 |
| C | 3.64241600 | 0.36925800 | 1.08353600 |
| H | 4.01799300 | 0.97401000 | 1.89724000 |
| C | 1.58006600 | 0.67028600 | −0.17003600 |
| C | 1.39619700 | −0.71291500 | −0.21113200 |
| C | 0.49632300 | 1.53338200 | −0.09986400 |
| C | −0.80463300 | 0.95933800 | −0.00203700 |
| C | −1.00674200 | −0.45330300 | −0.04978300 |
| C | 0.13185500 | −1.28375400 | −0.10504600 |
| C | −3.29965300 | 1.29303700 | 0.09938900 |
| H | −4.12504100 | 1.99299300 | 0.14272400 |
| C | −3.48276700 | −0.03988000 | 0.09866300 |
| H | −4.49250700 | −0.44051200 | 0.14867200 |
| C | −1.95968200 | 1.86894800 | 0.03442600 |
| C | 3.08000600 | 0.92212700 | −0.22635200 |
| H | 3.37764900 | 1.93045100 | −0.50315400 |
| C | 3.46746000 | −0.24785900 | −1.17095800 |
| H | 3.00496000 | −0.17312600 | −2.15721200 |
| H | 4.54706400 | −0.38478500 | −1.25502000 |
| C | 2.80047300 | −1.32037400 | −0.26524000 |
| H | 2.88295300 | −2.35872700 | −0.59003500 |
| C | −2.39529200 | −1.06554100 | 0.04094300 |
| C | −2.68791300 | −1.93754800 | −1.20165600 |
| H | −2.61074800 | −1.33390300 | −2.10771100 |
| H | −1.98953900 | −2.76823100 | −1.26717700 |
| H | −3.70288300 | −2.33735700 | −1.13843200 |
| C | −2.52859000 | −1.91235100 | 1.32781300 |
| H | −3.54015200 | −2.31971500 | 1.39748700 |
| H | −1.82003200 | −2.73728100 | 1.32089700 |
| H | −2.34486400 | −1.28944700 | 2.20491200 |

Compound **V**

E(MP2) = −843.76071778 a.u.

| | | | |
|---|---|---|---|
| C | −2.24075700 | −1.08607400 | −0.11253000 |
| O | 0.11567000 | −2.64451800 | 0.18396300 |
| H | 0.97361300 | −3.08759400 | 0.14054400 |
| O | 0.91784100 | 2.81067600 | −0.07867200 |
| H | 0.03292900 | 3.25285700 | −0.09597700 |
| C | −2.32930700 | −2.14789700 | −1.22354800 |
| H | −3.37871200 | −2.41826700 | −1.36898800 |
| H | −1.94992200 | −1.74265900 | −2.16443000 |
| H | −1.76984300 | −3.04626000 | −0.98137900 |
| H | −1.91290100 | −2.51504200 | 1.49423400 |
| C | −2.61294600 | −1.72110100 | 1.23810700 |
| H | −3.61733200 | −2.14972500 | 1.18124000 |
| H | −2.59913500 | −0.98382100 | 2.04287300 |
| O | −1.63380700 | 3.10556400 | −0.00402700 |
| C | 3.15713000 | 1.27934900 | −0.01531800 |
| H | 3.19528900 | 1.98065900 | −0.85457700 |
| H | 3.27908300 | 1.90546800 | 0.87625600 |
| C | 2.78360200 | −1.69270900 | 0.15631300 |
| H | 2.77010000 | −2.19005300 | 1.13630800 |
| H | 2.68711500 | −2.49170600 | −0.59241400 |
| C | −1.77325700 | 1.86502200 | 0.08223800 |
| C | 4.28180000 | 0.29660700 | −0.11161900 |
| H | 5.28011600 | 0.69955400 | −0.24545200 |
| C | 4.11730900 | −1.03254400 | −0.04013000 |
| H | 4.97944300 | −1.68669100 | −0.11212300 |
| C | 1.79712100 | 0.63554400 | 0.01569000 |
| C | 1.60955400 | −0.74798500 | 0.04867100 |
| C | 0.67211000 | 1.47267900 | −0.05536900 |
| C | −0.63720500 | 0.93640800 | −0.02414800 |
| C | −0.84257400 | −0.47457500 | −0.03415800 |
| C | 0.30053200 | −1.27753900 | 0.07495100 |
| C | −3.26813600 | −0.00442700 | −0.48239400 |
| H | −3.15023000 | 0.24908700 | −1.54203200 |
| H | −4.27196000 | −0.42363600 | −0.36744600 |
| C | −3.12470300 | 1.26707100 | 0.34001200 |
| H | −3.87933800 | 2.01447300 | 0.09237500 |
| H | −3.20757300 | 1.05261200 | 1.41027500 |

Compound **VI**

E(MP2) = −844.97057376a.u.

| | | | |
|---|---|---|---|
| C | 2.25260400 | −1.10165500 | 0.16801600 |
| O | −0.07202900 | −2.64301300 | −0.34772600 |
| H | −0.91966500 | −3.05316900 | −0.56516200 |
| O | −0.84702600 | 2.84078100 | −0.08497600 |
| H | 0.03933400 | 3.26617800 | 0.02297100 |
| C | 2.23578900 | −2.18056600 | 1.26607200 |
| H | 3.26659900 | −2.46343500 | 1.49652100 |
| H | 1.78107900 | −1.78528900 | 2.17735800 |
| H | 1.69197400 | −3.07069000 | 0.96471500 |
| H | 2.05290600 | −2.50263900 | −1.48276700 |
| C | 2.73561200 | −1.72028500 | −1.15511600 |
| H | 3.72719800 | −2.16018200 | −1.01746200 |
| H | 2.79868100 | −0.97077100 | −1.94607400 |
| O | 1.70060700 | 3.09503700 | 0.08093900 |
| C | −3.13976400 | 1.18180000 | −0.32849100 |
| H | −3.15269900 | 2.25364900 | −0.13697500 |
| H | −3.55810500 | 1.02605900 | −1.32998500 |
| C | −2.81730900 | −1.52896200 | −0.48658000 |
| H | −3.21111500 | −1.37003100 | −1.49654800 |
| H | −2.65909200 | −2.60664800 | −0.38890900 |
| C | 1.83106000 | 1.85415600 | −0.01566500 |
| C | −1.73305200 | 0.66234800 | −0.27435300 |
| C | −1.55489900 | −0.72480200 | −0.31720400 |
| C | −0.62225800 | 1.49908500 | −0.10894300 |
| C | 0.67849500 | 0.93900000 | −0.01985500 |
| C | 0.87083200 | −0.47564600 | −0.02183000 |
| C | −0.26111000 | −1.27611100 | −0.24210100 |
| C | 3.25498100 | −0.03797700 | 0.64202700 |
| H | 3.04916800 | 0.20151600 | 1.69148900 |
| H | 4.26078200 | −0.46670100 | 0.60707500 |
| C | 3.19355100 | 1.24586400 | −0.17027000 |
| H | 3.93190400 | 1.98189000 | 0.14993000 |
| H | 3.36267700 | 1.04539800 | −1.23299800 |
| C | −3.87844200 | −1.10554000 | 0.55048400 |
| H | −4.84013300 | −1.52665800 | 0.24850200 |
| H | −3.62249000 | −1.56301300 | 1.50825900 |
| C | −3.98083400 | 0.42810400 | 0.71264300 |
| H | −5.02273100 | 0.74949800 | 0.64995800 |
| H | −3.62023800 | 0.71971600 | 1.70120100 |

Compound **VII**

E(MP2) = −841.38495769a.u.

| | | | |
|---|---|---|---|
| C | 4.03065300 | −1.11064100 | −0.07625100 |
| C | 2.75058400 | −1.63560000 | −0.08305300 |
| C | 1.61159700 | −0.78762300 | −0.04323200 |
| C | 1.82500300 | 0.61982600 | 0.05936300 |
| C | 3.14286100 | 1.14007100 | 0.04803700 |
| C | 4.22868000 | 0.28498000 | 0.01773200 |
| C | 0.27970000 | −1.29517000 | 0.02449500 |
| C | 0.69678200 | 1.48569300 | 0.01608400 |
| C | −0.60337000 | 0.95862700 | 0.02428200 |
| C | −0.83047800 | −0.46126300 | −0.01283100 |
| C | −2.23973800 | −1.03754200 | 0.00716500 |
| C | −3.29810200 | 0.02096800 | −0.05048000 |
| C | −3.08507800 | 1.34941500 | −0.07290800 |
| C | −1.73241200 | 1.89566300 | −0.03874300 |
| H | 4.88346200 | −1.77662800 | −0.12841000 |
| H | 2.63680500 | −2.70711600 | −0.20301700 |
| H | 3.27666600 | 2.21232800 | 0.09953400 |
| H | 5.23426700 | 0.68734500 | 0.02209700 |
| H | −4.31917500 | −0.35281700 | −0.06978700 |
| H | −3.89606900 | 2.06616800 | −0.11268200 |
| O | 0.08143700 | −2.66658100 | 0.01727700 |
| H | 0.85183100 | −3.09002700 | 0.42076300 |
| O | 0.95160500 | 2.81385100 | 0.04168700 |
| H | 0.06268300 | 3.26008100 | 0.01281600 |
| C | −2.47987500 | −1.94866500 | −1.21767400 |
| H | −3.50928800 | −2.31532200 | −1.20475400 |
| H | −2.32727600 | −1.38435100 | −2.13934600 |
| H | −1.80639100 | −2.80205100 | −1.20943800 |
| H | −1.81053000 | −2.67456600 | 1.38004600 |
| C | −2.48666600 | −1.82545200 | 1.31359400 |
| H | −3.51513100 | −2.19417100 | 1.33406700 |
| H | −2.33461100 | −1.17560800 | 2.17704800 |
| O | −1.56905200 | 3.14360500 | −0.04301600 |

Compound **VIII**

E(MP2) = −992.43761434 a.u.

| | | | |
|---|---|---|---|
| C | 3.59518100 | −0.88821200 | 0.00293300 |
| C | 2.28512700 | −1.32734700 | 0.00528000 |
| C | 1.25099200 | −0.38996500 | −0.05389100 |
| C | 1.42109500 | 1.00016100 | 0.02246500 |
| C | 2.77102100 | 1.46783300 | −0.00090700 |
| C | 3.79805300 | 0.51268600 | 0.02431200 |
| C | 0.02257700 | −1.05775200 | −0.00482500 |
| C | 0.19698900 | 1.74257600 | −0.01852300 |
| H | 4.43364200 | −1.57385000 | 0.03080600 |
| H | 4.81887900 | 0.87850000 | 0.02262900 |
| O | 0.23357200 | −2.43306300 | −0.01685800 |
| O | 0.27784600 | 3.08994300 | −0.01631900 |
| H | −0.66566900 | 3.41944700 | −0.02736100 |
| C | −2.62632600 | −2.02189000 | −1.21284200 |
| H | −3.59069700 | −2.53488100 | −1.19432300 |
| H | −2.55963100 | −1.44067300 | −2.13393900 |
| H | −1.83509200 | −2.77068200 | −1.20705300 |
| H | −1.81736700 | −2.69379700 | 1.32520700 |
| C | −2.60185700 | −1.93738400 | 1.30714100 |
| H | −3.57066200 | −2.44039400 | 1.35060700 |
| H | −2.50077100 | −1.29816600 | 2.18563500 |
| O | −2.24803900 | 3.13357900 | −0.03194200 |
| C | 1.64322400 | −2.66203000 | −0.01886500 |
| O | 2.09521900 | −3.78502600 | −0.02927300 |
| C | 3.11779100 | 2.93218500 | 0.02552700 |
| H | 2.70885400 | 3.44739900 | −0.84306500 |
| H | 2.69578100 | 3.41686200 | 0.90536500 |
| H | 4.19999600 | 3.05982500 | 0.03538400 |
| C | −2.27381600 | 1.87497700 | −0.01744900 |
| C | −3.56657000 | 1.19119800 | −0.02020800 |
| H | −4.44088700 | 1.83036200 | −0.03623700 |
| C | −3.66168000 | −0.15228600 | −0.00582800 |
| H | −4.64455600 | −0.61745500 | −0.00544700 |
| C | −1.17246000 | −0.37521800 | −0.01799200 |
| C | −1.04737000 | 1.06522500 | 0.02528700 |
| C | −2.50207200 | −1.09575100 | 0.01620500 |
